# Are Electronic Notifications in Imaging Order Communication Systems an Effective Means of Changing Clinicians' Behaviour?

**DOI:** 10.7759/cureus.31378

**Published:** 2022-11-11

**Authors:** Amjad Burgan

**Affiliations:** 1 Department of Trauma and Orthopaedics, Yeovil District Hospital NHS Foundation Trust, Yeovil, GBR

**Keywords:** ottawa ankle rules, order communication system, electronic clinical decision support system, ankle trauma, x-ray analysis

## Abstract

Introduction

Order Communication Systems (Ordercomms) are computer applications used to enter diagnostic and therapeutic patient care orders and view test results. These electronic systems allow the integration of Clinical Decision Support Systems (CDSS). CDSS are computer applications designed to aid clinicians in making diagnostic and therapeutic decisions in patient care (e.g. can notify clinicians of best practice guidelines when requesting investigations or prescribing medications). The aims of this study were to determine whether electronic notifications (via Ordercomms) are effective in improving clinician compliance with the Ottawa Rules in plain radiographs requesting for ankle trauma, and the efficacy of electronic notifications in reducing inappropriate imaging requests.

Methods

The Ottawa Rules are a globally validated clinical decision tool with a sensitivity of 99%-100% for ankle fractures. When used, they can reduce the number of unnecessary radiographs by 30%-40%. Importantly, the Royal College of Radiologists stipulates that a patient must fulfill the Ottawa Rules in order to proceed with a plain radiograph of the ankle in trauma. A retrospective analysis of 366 plain ankle radiographs was performed to exclude bony injury in the emergency department between February and March 2018. Information gathered included patient demographics, the request form completed by the emergency department clinician, and radiology report. A pop-up reminder was then implemented on the electronic requesting system to prompt clinicians to apply the Ottawa Ankle Rules and document their plain radiograph request accordingly. Following the intervention, a further 473 plain radiographs were analysed in the same way over a three-month period (April-June 2018).

Results

In the two months prior to the intervention, 366 plain radiographs were performed for ankle trauma. Of these, 45.1% fulfilled the Ottawa Rules. In the three months following our intervention, 473 plain radiographs were carried out. There was no significant increase in the percentage of requests fulfilling the Ottawa Rules (45.7%). Unnecessary radiographs (those which did not fulfill the Ottawa Rules and consequently showed no fracture) also showed no change. The data demonstrates that the electronic reminder asking individuals to apply and document the Ottawa Rules appropriately had no impact on the imaging requesting behaviour, and subsequently on the number of unnecessary plain radiographs.

Conclusion

Electronic notifications in Order Communication Systems did not change clinicians' behaviour in this specific circumstance. This study has implications for electronic notifications in prescribing systems and pathology requesting systems. Further research is needed to determine if the findings are replicated with other imaging types.

## Introduction

Order Communication Systems (Ordercomms) are computer applications used to enter diagnostic and therapeutic patient care orders and view test results [1]. Their use in modern healthcare has expanded rapidly with clear benefits to clinicians, patients and administrative staff in safety and efficiency. These electronic systems have the added benefit of enabling the integration of Clinical Decision Support Systems (CDSS). CDSS are computer applications designed to aid clinicians in making diagnostic and therapeutic decisions in patient care [2]. A great number of NHS trusts have implemented Ordercomms and CDSS alongside picture archiving and communication systems (PACS), and in 2022, the Royal College of Radiologists (RCR) published guidelines on the implementation of Ordercomms [3]. While the argument for CDSS is compelling, there is mixed evidence regarding its efficacy in published systematic reviews [4]. Research into the effect of CDSS in the context of imaging requesting is even more limited. To our knowledge, only one systematic review has been published, with inconclusive results, and varying outcomes dependent on imaging modality, study methodology and measured outcome [5]. CDSS, in their most simple form, can notify clinicians of best practice guidelines when requesting investigations or prescribing medications.

The objective here was to establish whether electronic notifications (via Ordercomms) are effective in improving clinician compliance with the Ottawa Rules in plain radiographs requesting for ankle trauma, and to determine the efficacy of electronic notifications in reducing inappropriate imaging requests.

## Materials and methods

This single-centre prospective interventional study was performed in the emergency department of a district general hospital in the South of England, serving a population of 380,000 people [6]. Using the Ordercomms system, we retrospectively analysed 366 plain ankle radiographs performed to exclude bony injury between February and March 2018. Information gathered included patient demographics, the request form completed by the emergency department clinician, and radiology report. The request forms were reviewed for inclusion of the Ottawa Ankle rules (malleolar pain and weight bearing status) and the indication for the plain radiograph. Only plain radiographs requested to exclude fractures were included in the final analysis. A pop-up reminder was then implemented on the electronic requesting system to prompt clinicians to apply the Ottawa Ankle Rules and document their plain radiograph request accordingly. This reminder stated, "Please document if: Malleolar pain, weight bearing". The pop-up reminder was activated for all ankle plain radiographs requested from the emergency department. Following the intervention, a further 473 plain radiographs were analysed in the same way over a three-month period (April-June 2018). Unnecessary radiographs were calculated by determining the false negatives, i.e. where requests did not fulfill the clinical decision tool and radiographs showed no fracture. The research was registered and gained clinical approval from the hospital audit department. Ethical approval was not required.

The Ottawa Rules are a globally validated clinical decision tool with a sensitivity of 99%-100% for ankle fractures [7,8]. When used, they can reduce the number of unnecessary radiographs by 30%-40% [9]. Importantly, the RCR stipulates that a patient must fulfill the Ottawa Rules in order to proceed with a plain radiograph of the ankle in trauma [10]. The Ottawa Rules for ankle trauma state that a patient should only have an x-ray of their ankle if they present with tenderness of the malleolar zone and either bony tenderness at the posterior edge or tip of the medial or lateral malleolus, or an inability to weight bear for four steps (both immediately and in the emergency department).

## Results

In the two months prior to the intervention, 366 plain radiographs were performed for ankle trauma. Of these plain radiograph requests, 45.1% fulfilled the Ottawa Rules. Of the 366 plain radiographs, 42.9% did not fulfill the Ottawa Rules and subsequently revealed no ankle fracture and were therefore unnecessary.

In the three months following our intervention, 473 plain radiographs were carried out. Of these, 216 requests fulfilled the Ottawa Rules. There was no increase in the percentage of requests fulfilling the Ottawa Rules (45.7%). Unnecessary radiographs (those which did not fulfill the Ottawa Rules and consequently showed no fracture) also showed no change (40.8%) (Table 1).

**Table 1:** Plain ankle radiograph requests fulfilling the Ottawa Rules and unnecessary plain ankle radiographs before and after the implementation of an electronic notification

	Prior to the intervention	Following the intervention
Total cases	366	473
Requests fulfilling Ottawa Rules	165	216
Requests not fulfilling Ottawa Rules	201	257
Percentage of requests fulfilling Ottawa Rules (%)	45.1	45.7
Percentage of unnecessary radiographs (%)	42.9	40.8

## Discussion

This data demonstrates that the electronic reminder asking individuals to apply and document the Ottawa Rules appropriately had no impact on imaging requesting behaviour, and subsequently, on the number of unnecessary plain radiographs. This fact raises the question, "Why did clinician behaviour remain unchanged"? It is clear from our results that the Ottawa Rules were not implemented in the accident and emergency (A&E) department even prior to the intervention, despite the RCR stipulation. The Ottawa Rules are a useful tool in reducing the pressure on radiology departments, which is of paramount importance in the context of the current demands on the National Health Service [11]. It may be that clinicians are using the Ottawa Rules but not documenting it in the requests. Other reasons may include the relatively low cost and availability of plain radiograph imaging in the emergency department, patient and clinician reassurance and low risk of a single plain radiograph. However, if we accept the results of the study, whereby the only variable was the presence or absence of an electronic notification, then the inefficacy of these notifications may also be a part of the reason why no change in behaviour was observed. This implies that while these electronic notifications may seem like a cheap and easy way to change clinical practice for the better, in reality, there may need to be greater engagement with a clinician than a simple pop-up notification.

Published research has highlighted the benefits of Ordercomms including reduced paperwork, reliable electronic records and almost instantaneous communication, leading to greater efficiency and safety of healthcare provision [12]. There is also published evidence that CDSS can improve adherence to radiology requesting guidelines; however, the majority of these studies are of limited scope and with differing outcomes [1,13]. For example, published data on the use of CDSS to influence clinician requests for neuroradiology is reported to be more effective than requesting of plain abdominal or chest radiographs [14,15]. This suggests that a clinician's response to CDSS is dependent on multiple factors and may go some way to explaining why there was a limited response to the intervention in this study. Access to plain radiograph imaging is available in almost every emergency department and minor injury unit, and therefore, ease of access may lower the clinical threshold for requesting a plain radiograph. Further research of the reasons for the absence of behavioural change in response to the electronic notification is beyond the scope of this study.

There are some clear limitations of this study. It was a single-centre study with a narrow scope, assessing only one imaging modality in a specific setting. The study was also limited by a low absolute number of cases. There was also no investigation of the causes behind the lack of the impact of the intervention that was beyond the scope of this study. It also did not capture imaging requests that were not made as a result of the pop-up; however, the number of requests per month did not reduce following the intervention, suggesting this did occur in substantial numbers. The primary strength was in the study design. There was only one variable (the presence or absence of a notification), and therefore, the measured outcome is a reliable indicator of the impact of this change.

## Conclusions

In conclusion, electronic notifications in Order Communication Systems did not change clinicians' behaviour in this specific circumstance. This study adds to the body of research in a field with limited published data. Determining the course of best practice not only relies on the demonstration of efficacy but also the identification of inefficacy. More research is required in this field to determine if these findings are replicated with other imaging types and if targeted electronic interventions are more effective.

This study has implications for electronic notifications in prescribing systems and pathology requesting systems. It is clear that despite limited evidence, CDSS is already commonplace and its use will only increase as digital medical technologies replace the paper notes of the past. In this ever-progressing field, it is essential to determine if the changes we make have the desired outcome.
